# Long-term efficacy and safety of omalizumab in patients with persistent uncontrolled allergic asthma: a systematic review and meta-analysis

**DOI:** 10.1038/srep08191

**Published:** 2015-02-03

**Authors:** Tianwen Lai, Shaobin Wang, Zhiwei Xu, Chao Zhang, Yun Zhao, Yue Hu, Chao Cao, Songmin Ying, Zhihua Chen, Wen Li, Bin Wu, Huahao Shen

**Affiliations:** 1 Department of Respiratory and Critical Care Medicine, Second Affiliated Hospital, Institute of Respiratory Diseases, Zhejiang University School of Medicine, Hangzhou, China; 2 Department of Respiratory and Critical Care Medicine, Affiliated Hospital, Institute of Respiratory Diseases, Guangdong Medicine College, Zhanjiang, China; 3 State Key Lab. for Respiratory Diseases, Guangzhou, China; 4 Department of Pharmacology, Zhejiang University School of Medicine, Hangzhou, China

## Abstract

Currently, limited information is available to clinicians regarding the long-term efficacy of omalizumab treatment for allergic asthma. In this report, we aimed to (i) systematically review the evidence regarding the long-term efficacy of omalizumab in patients with persistent uncontrolled allergic asthma, and to (ii) discuss the cost-effectiveness evidence published for omalizumab in this patient population. A comprehensive search for randomized controlled trials (RCTs; ≥52 weeks) was performed, and six studies met our final inclusion criteria (n = 2,749). Omalizumab was associated with significant improvements in quality of life and the Global Evaluation of Treatment Effectiveness. Omalizumab also allowed patients to completely withdraw from inhaled corticosteroid therapy and did not increase the overall incidence of adverse events. However, there was insufficient evidence that omalizumab reduced the incidence of exacerbations, and the cost-effectiveness of omalizumab varied across studies. Our data indicated that omalizumab use for at least 52 weeks in patients with persistent uncontrolled allergic asthma was accompanied by an acceptable safety profile, but it lacked effect on the asthma exacerbations. Use of omalizumab was associated with a higher cost than conventional therapy, but these increases may be cost-effective if the medication is used in patients with severe allergic asthma.

Asthma is characterized by bronchial inflammation, airway hyper-responsiveness induced by specific and nonspecific stimuli, and reversible bronchial obstruction^1^,^2^,^3^. An estimated 57% of these asthma patients suffer from uncontrolled asthma and a substantial proportion of severe cases are attributable to allergic immunoglobulin E (IgE)-mediated mechanisms^4^,^5^,^6^,^7^,^8^. Patients with persistent uncontrolled asthma are at high risk of asthma-related hospitalization and mortality, suffer significant impairments in their quality of life (QOL), and account for the majority of asthma-related costs. The Global Initiative for Asthma (GINA) guidelines recommend a stepwise approach to asthma control, with treatment being stepped up until control is achieved and maintained. However, even with the availability of these asthma guidelines and the best available treatments, approximately one third of patients continue to suffer from inadequately controlled symptoms. For patients whose asthma remains uncontrolled at this step, GINA recommends adding oral corticosteroids (OCS) or anti-IgE treatment with omalizumab^9^. However, adding OCS is associated with severe side effects. Specific targeting of IgE with an anti-IgE antibody therefore represents a promising approach to the treatment of allergic asthma^10^,^11^,^12^. Omalizumab is a recombinant humanized IgG1 monoclonal anti-IgE antibody that binds IgE at the same epitope on the Fc region that binds to the IgE receptor^13^,^14^,^15^.

Although omalizumab is an effective intervention as an add-on therapy in the management of severe persistent allergic asthma, important questions remain regarding the role of omalizumab in the treatment of asthma based on current guidelines. Updated National Institute for Health and Care Excellence (NICE 2013) guidelines recommend use only in patients with inadequately controlled severe persistent allergic asthma who require continuous or frequent treatments with oral corticosteroids^16^. However, this recommendation is not strongly supported by evidence. Indeed, other international guidelines are less proscriptive and recommend this treatment for patients who remain suboptimally controlled after maximal therapy with inhaled corticosteroids (ICS) plus long-acting beta2-agonists (LABA), as well as a third controller (e.g., leukotriene antagonists or theophyllines)^16^. Furthermore, evidence is somewhat lacking regarding the efficacy of this drug in patients with more severe asthma, as many trials include participants with mild or moderate disease^16^. In the US, omalizumab is recommended for the treatment of adults and adolescents (aged 12 years and above) with moderate-to-severe allergic asthma that is inadequately controlled in spite of treatment with ICS. This approval was based on previous pivotal clinical trials that did not include patients using LABAs, as these trials were designed and implemented at a time when LABAs were not the standard of care for asthma. Over time, LABAs have become the standard of care for patients with asthma that is not adequately controlled with ICS therapy^17^. The updated asthma treatment guidelines recommend omalizumab as an add-on treatment for steps 5 and 6 and include high doses of ICS and LABA combination therapy (with OCS added at step 6). However, little evidence has been found for this recommendation^9^,^18^. Omalizumab treatment efficacy is often evaluated at 16 weeks; however, in many patients, an extension of treatment is essential to improve symptoms, medication use, lung function and quality of life outcomes. For this reason, when to stop omalizumab therapy, as well as its long-term effects, are unclear. Long-term studies will be needed to clarify these issues. In 2009, the US Food and Drug Administration (FDA) raised concerns about the incidence of adverse cardiovascular and cerebrovascular events in the omalizumab treatment group of the EXCELS study^19^. Such events were not described in previous analyses of clinical data, and several systematic reviews have not observed increased cardiovascular risk among patients taking omalizumab in studies shorter than 1 year^16^,^17^,^20^,^21^,^22^. The FDA is not recommending any changes in the drug's prescription information at this time. The long-term (more than 1 year) efficacy and safety of omalizumab remain a concern.

Numerous randomized clinical trials (RCTs) have demonstrated omalizumab's efficacy in patients with moderate-to-severe allergic asthma, but treatment periods have always been relatively short in these trials (mean: 28 [range: 20 to 32] weeks). We have summarized the main characteristics of those studies in supplemental table 1^23^,^24^,^25^,^26^,^27^,^28^,^29^,^30^,^31^,^32^,^33^,^34^,^35^,^36^,^37^,^38^,^39^,^40^,^41^,^42^. Because the value of short-term treatment outcomes is relatively limited, assessments covering longer periods of treatment are necessary. As asthma is a chronic disease, long-term studies are necessary to evaluate the effects of omalizumab therapy, especially in children. There is a lack of robust evidence regarding the efficacy of omalizumab beyond 52 weeks in both adults and children. In recent years, several RCTs have assessed the effects of long-term (≥52 weeks) omalizumab treatment in patients with allergic asthma. However, the evidence is inadequate for drawing robust conclusions, because the sample sizes of these studies were relatively modest and their conclusions were inconsistent. To comprehensively evaluate the evidence relating to these issues, we conducted this study to determine whether omalizumab is safe and effective when used for more than 52 weeks in patients with persistent, uncontrolled, moderate-to-severe allergic asthma in spite of high-dose ICS or ICS plus LABA, and to provide clinicians with evidence regarding the long-term efficiency of omalizumab treatment in patients with allergic asthma. Additionally, omalizumab is more expensive than other asthma treatments, and evidence of economic benefits for patients and reimbursement authorities remains in demand^43^. Therefore, the cost-effectiveness evidence published for omalizumab in this patient population was also examined.

## Results

### Characteristics of the studies

**Figure 1:**
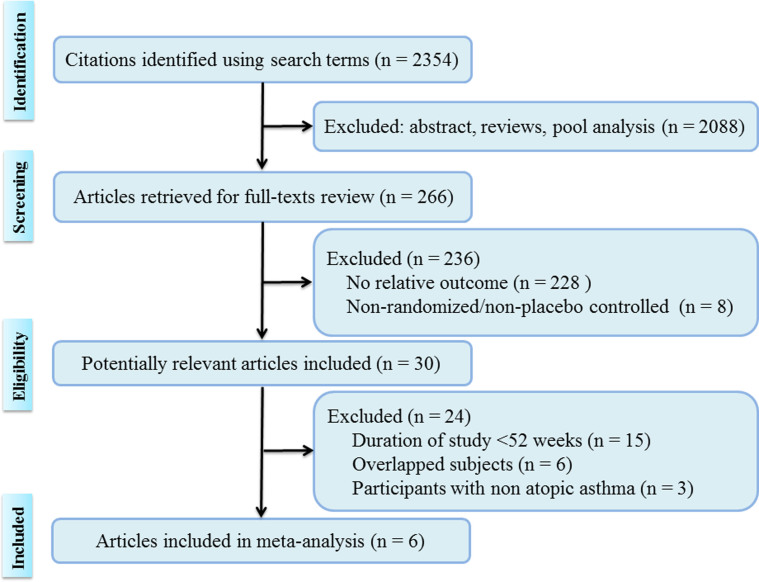
Flow diagram.

The electronic database search identified 2,354 citations. Of these, the first screening excluded 2,088 citations based on abstracts or titles, leaving 266 articles for full-text review. Of these articles, 236 studies were excluded because they contained no relative outcomes or were non-randomized, or non-placebo controlled studies. Following a more detailed review, fifteen short term trials (<52 weeks) were excluded. Finally, six studies were included in our systematic review and meta-analysis^44^,^45^,^46^,^47^,^48^,^49^. The detailed steps of the study selection process are shown in Figure 1.

The primary characteristics of the included studies are summarized in Table 1. A total of 2,749 participants with allergic asthma were included in these studies. Patients with severe asthma were recruited in three studies^44^,^45^,^46^, and patients with moderate-to- severe asthma were recruited in three additional studies^47^,^48^,^49^. Mean baseline FEV_1_ values varied from 64.1 to 92.9% of predicted. Durations of treatment ranged from 52 to 62 weeks. Four studies included adolescents and adults only^44^,^45^,^46^,^47^, and two also included pediatric paticipants^48^,^49^. Regarding the risk of bias, only one study met all five accepted criteria^48^ (Table 2).

### Treatment effectiveness and safety

**Figure 2:**
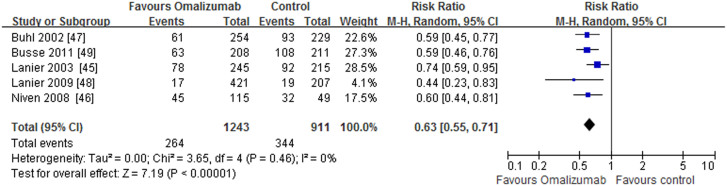
The effect of omlizumab on asthma exacerbations.

**Figure 3:**
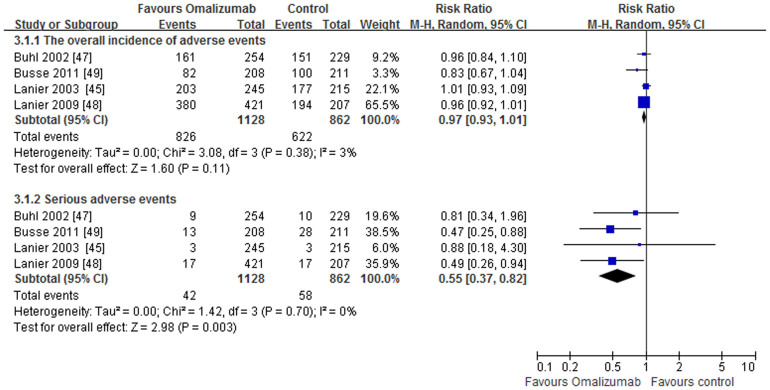
The effect of omlizumab on the adverse events.

The overall designs of these studies were as follows: after a run-in phase (4–8 weeks), omalizumab was administrated as an adjunctive therapy to inhaled or oral corticosteroids for 16 to 28 weeks (stable steroid phase), followed by a steroid-reduction phase of 12 to 28 additional weeks, during which doses were decreased only if patients met strict criteria for steroid reduction. We double-counted two end points (stable steroid phase and steroid-reduction phase), and using these single primary efficacy endpoints (end of the steroid-reduction phase), included the rates of clinically significant asthma exacerbations, reductions in ICS doses, Global Evaluation of Treatment Effectiveness (GETE), Asthma Quality of Life Questionnaire (AQLQ), asthma symptom scores, lung function, and adverse events (AEs), over a period of 52 weeks. Although all studies included a steroid reduction phase, only two reported data regarding a stable steroid phase^44^,^48^. The data showed that omalizumab-treated patients experienced significantly lower rates of clinically significant asthma exacerbations compared with patients who received a placebo during the stable phase (0.45 vs 0.64; p = 0.007), and the relative risk (RR) was 0.69 [0.53, 0.90]. Data from the studies with a steroid-reduction phase demonstrated reductions in exacerbation rates that remained significant over periods of 52 weeks (RR 0.63, 95% CI [0.55, 0.71]; p < 0.0001) (Figure 2). Statistical heterogeneity was not observed (I^2^ = 0%, p = 0.46). During the steroid-reduction phase, ICS doses were significantly decreased in omalizumab-treated patients compared with the placebo group (RR 1.86, 95% CI [1.51, 2.29]; p < 0.0001). Heterogeneity was not observed (I^2^ = 0%, p = 0.47). At 52 weeks, both GETE (an excellent or good response) and AQLQ scores (≥1.5 points from baseline) favored omalizumab (RR1.54, 95%CI [1.38, 1.72]; p < 0.00001 and RR 2.08, 95% CI [1.03, 4.20]; p = 0.04 respectively) (table 3). Four studies assessed adverse events (AEs), and omalizumab was well tolerated^45^,^47^,^48^,^49^. Common adverse events included the following: lower respiratory tract infection, nasopharyngitis, headache, injection site pain, injection site reaction and arthralgia. Based on the results of the meta-analysis, the numbers of patients reporting AEs was similar in both treatment groups (RR 0.97, 95% CI [0.93, 1.01]; p = 0.11). Statistical heterogeneity was not observed (I^2^ = 3%, p = 0.38). Serious adverse events, such as death, asthma exacerbation, pruritus, acute appendicitis, sphenoid sinusitis, intestinal obstruction, and mild chest pain were reported. However, none of these was considered drug-related. The incidence and profile of serious adverse events were slightly lower in the omalizumab group (RR 0.55, 95% CI [0.37, 0.82]; p = 0.003). Statistical heterogeneity was not observed (I^2^ = 0%, p = 0.70) (Figure 3). No clinically relevant abnormalities in laboratory tests (including platelet count) were observed.

With regard to asthma symptoms and lung function, descriptive analysis methods were utilized, as most of these data were unavailable or unsuitable for analysis. Two RCTs demonstrated greater reductions in asthma symptom scores than placebo^46^,^48^. However, the effects of omalizumab on lung function were discrepant^45^,^46^,^47^,^49^. Only one RCT demonstrated that pulmonary function (FEV_1_) was significantly better in the omalizumab group than in the control group^46^ (Supplemental table 2).

The cost-effectiveness of omalizumab add-on therapy has been assessed in several analyses^43^,^50^,^51^,^52^,^53^,^54^,^55^,^56^,^57^. Marked variations were noted across studies regarding cost-effectiveness (Table 4). Campbell et al. concluded that adding omalizumab improves quality-adjusted life years (QALY), with increased direct medical costs^51^. Their findings also suggested that cost-effectiveness improves when 16-week assessments to determine responses are used to guide decisions regarding long-term treatment. Dal Negro et al. concluded that omalizumab improves health-related quality of life but also substantially increases costs^53^. Nooten and Wu et al. reported that omalizumab was not cost-effective and noted incremental cost-effectiveness ratios (ICERs) of [euro]38,371 and $821,000^54^,^56^. Data from the real-life 1-year randomized open-label study (ETOPA), using Canada as a reference country, noted an ICER of [euro]31,209 in patients with severe persistent allergic asthma^50^. Devilde and Oba et al. concluded that omalizumab was cost-effective for patients with severe allergic asthma^52^,^55^. This finding suggests that asthma severity and the risk of asthma exacerbations should be considered when determining the cost-effectiveness of omalizumab.

### Sensitivity analysis and publication bias

Two outcomes (asthma exacerbations and AQLQ) were analyzed in an open label study^46^. When only randomized, double-blind trials were evaluated^44^,^45^,^47^,^48^,^49^, there were no significant differences in the incidences of asthma exacerbations (RR 0.63, 95% CI [0.54, 0.73]; p < 0.00001). AQLQ was also not significantly altered when an open label study was excluded (RR1.57, 95% CI [1.23, 2.01]; p = 0.0003). Other sensitivity and subgroup analyses are summarized in table 4. A sensitivity analysis revealed that the conclusions of the meta-analysis remained robust regarding methodological changes, indicating that the results of our study are believable and reliable. Publication bias was detected by Begg's and Egger's tests. Funnel plots of the four studies evaluating the effects of omalizumab on asthma exacerbation appeared to be symmetrical upon visual examination. The data suggested that there was no evidence of publication bias (Begg's test, p = 0.373, Eger's test, p = 0.568).

## Discussion

Severe persistent asthma remains poorly understood and difficult to manage. Previous studies and reviews have demonstrated that omalizumab is an effective treatment option for moderate to severe allergic asthma. However, evidence regarding its long-term (beyond 52 weeks) efficacy and safety in both adults and children is very limited. In recent years, new randomized trials have assessed the efficacy and safety of omalizumab beyond 52 weeks in both adults and children with allergic asthma that is poorly controlled in spite of treatment with high doses of ICS or ICS plus LABA. Therefore, it seems reasonable to explore this issue further. In contrast to previous systematic reviews that included studies of short duration (less than 1 year), we included only long-term trials involving patients with persistent uncontrolled allergic asthma to assess the efficacy of and risk associated with omalizumab. Based on the pooled analyses, we found that omalizumab significantly reduced the incidence of asthma exacerbations and ICS use and improved scores on the GETE and AQLQ, compared with control subjects. Additionally, omalizumab was well tolerated and demonstrated an acceptable safety profile. Costs also increased, but the drug may be cost-effective if used in patients with severe allergic asthma.

Severe asthma exacerbations are a major concern, as they are responsible for the mortality associated with asthma and contribute significantly to the health costs of the disease^58^. Indeed, decreasing the rate of asthma exacerbations is a key goal of asthma management and is likely to be associated with improvements in asthma-related quality of life and reductions in the burdens imposed on patients and health care systems. Our meta-analysis demonstrated that compared with the control group, a significant reduction was observed in the rate of exacerbation for patients receiving omalizumab add-on treatment. However, there was some degree of heterogeneity in the definition of exacerbations within trials (Table 1), which may influence the efficacy of omalizumab on asthma exacerbations. Moreover, in placebo controlled study if you reduce treatment you will see more exacerbations allied to treatment dose reduction (e.g., steroid, LABA or a third controller). Therefore, the results should be interpreted cautiously due to these limitations. In other words, a lack of robust evidence existed that omalizumab reduced exacerbations in allergic asthma patients who were uncontrolled by the best available therapy. Our analyses demonstrated that the safety profile of omalizumab was excellent, and the treatment was well tolerated, as only infrequent and generally mild local reactions were observed following treatment. There were no drug-related serious adverse events. ICS are anti-inflammatory medications that inhibit inflammatory cell migration and activation, reduce airway hyperresponsiveness, and block late phase reactions to allergens^44^. ICS are fundamental in the treatment of asthma and are well tolerated and safe when administered at recommended dosages. In our meta-analysis, omalizumab-treated patients were more likely to be completely withdrawn from corticosteroid therapy. Reductions in ICS doses were achieved without precipitating worsening symptoms, increasing the use of rescue medications, altering lung function, or causing asthma exacerbations.

The GETE is a composite measure that includes patient interviews, reviews of medical notes, spirometry and diaries of symptoms, rescue medication use, and peak expiratory flow (PEF) values^59^. Our result demonstrated that more patients in the omalizumab group were rated as excellent or good compared with the control group by this measure. The emotional, physical and social aspects of the daily lives of patients with persistent uncontrolled allergic asthma are significantly impaired^3^. In our study, improvements in AQLQ overall scores (symptoms, activities, environment, and emotions) occurred in a larger proportion of patients receiving add-on omalizumab therapy, as these patients experienced significant (≥1.5-points) improvements in asthma-related QOL compared with the control group. This result was consistent with those of previous meta-analyses completed by Chipps et al. and Niebauer et al. (both in 2006)^60^,^61^.

Our study also demonstrated significant improvements in asthma symptom scores for omalizumab treatment compared with placebo. However, the effects of omalizumab on lung function were inconsistent. Several studies indicated that lung function parameters were not sensitive enough to mirror the treatment effectiveness of omalizumab in patients with asthma^45^,^46^,^47^,^49^. The improvements in lung function with omalizumab were consistent with its anti-inflammatory effects, improvements that may be attributed to suppressed free IgE levels^62^,^63^. The effects of omalizumab on lung function were particularly notable when placebo-treated patients received higher doses of long-acting bronchodilators, as they would be expected to diminish or eliminate any differences between the groups regarding lung function^62^. Seven studies were considered in this review and provided evidence of the cost-effectiveness of add-on omalizumab in patients with allergic asthma that was poorly controlled in spite of high doses of ICS or ICS plus LABAs. Although the findings of some economic analyses of omalizumab are unfavorable, there are published cost-effectiveness analyses that demonstrate that omalizumab is cost-effective in patients with inadequately controlled severe allergic asthma. Based on the high cost of omalizumab, it is important to determine which patients benefit most from its use.

There are some limitations that need to be considered. First, we included an open-label study under the assumption that real-world effectiveness data are meaningful and avoid the biases inherent in selecting studies. Although not as scientifically rigorous as double-blind trials, these types of trials remain important in understanding how a therapy performs in a setting more reflective of the real world^46^. However, the limitations of such trial designs (e.g., the potential for bias in the assessment of outcomes) should be considered when interpreting open-label data, which should be considered in the context of other randomized, double-blind data. To reconcile these issues, a sensitivity analysis was conducted whereby the meta-analysis was reanalyzed, excluding this open label study, as described in previous studies^64^,^65^,^66^. The sensitivity analysis determined that the conclusions remained robust for methodological changes, demonstrating that the data of present study are reliable. Second, some forms of detailed information (e.g., lung function and rescue medications) were unavailable in most studies, which prevented us conducting more detailed and relevant analyses and obtaining more comprehensive results. Therefore, the effects of omalizumab on lung function and rescue medications warrant further investigation.

The major strength of our study was that we included only long-term trials that provided evidence regarding the long-term efficacy and safety of omalizumab treatment in patients with uncontrolled persistent allergic asthma. Our study is in line with current guidelines, which recommend that omalizumab be considered as part of steps 5 and 6 of the stepwise treatment for patients with persistent allergic asthma that is uncontrolled in spite of treatment with high-dose ICS or ICS plus LABAs and/or a third controller (including OCS)^9^. Previous Cochrane reviews have consistently observed that omalizumab is both effective and safe in patients with inadequately controlled persistent asthma compared with conventional therapy^16^,^20^, and this conclusion is strengthened by our findings.

In summary, our findings have the following potential regulatory and clinical implications: 1) the use of omalizumab for at least 52 weeks in severe asthmatic patients is effective and is accompanied by an acceptable safety profile; 2) subgroup analyses provided further evidence for the current asthma guideline recommendations to consider omalizumab in steps 5 or 6 for patients with persistent allergic asthma that remains uncontrolled in spite of treatment with high-dose ICS plus LABAs and/or a third controller (including OCS); 3) Although omalizumab is often prescribed to reduce exacerbations, it lacks effect on exacerbations in patients with persistent uncontrolled allergic asthma; 4) costs increased, but the use of omalizumab could be cost-effective if the drug is used to treat patients with severe allergic asthma. However, the evidence in children is weaker and more ambiguous. Further studies are necessary to answer several practical questions, including how and when to reduce or stop treatment and how to identify possible genetic or biochemical markers that can predict treatment responses.

## Methods

### Data Sources and Search Strategy

We searched Medline, the Cochrane Database of Systematic Reviews (CDSR), EMBASE databases, the Cochrane Central Register of Controlled Trials (CENTRAL), the National Institutes for Health (NIH) ClinicalTrials.gov Register, Current Controlled Trials, and the FDA (www.fda.gov) database, which include all papers published up until March 2014, using the following search terms: “anti-immunoglobulin E” or “anti-IgE” or “Omalizumab” or “Xolair” and “asthma”. Trials were not excluded on the basis of language. All eligible studies were retrieved, and their reference lists were checked for additional articles. To ensure a complete review of the available studies, the abstracts of relevant scientific meetings were also examined. We also made efforts to contact authors in cases where relevant data were unclear. Trials published solely in abstract form were excluded.

### Selection criteria

Specific inclusion criteria were as follows: (1) adults/adolescents (12 years or older) and children (aged between 6 and 12 years) with a diagnosis of persistent uncontrolled moderate to severe allergic asthma in spite of high-dose ICS or ICS plus LABAs, (2) investigations of patients who received subcutaneous omalizumab therapy at any dose as a guidelines-based therapy, (3) randomized (parallel group) placebo-controlled trials, and (4) RCTs that reported the following outcomes: asthma exacerbations, inhaled corticosteroid (ICS) use, Evaluation of Treatment Effectiveness (GETE), QOL, asthma symptoms, lung function, rescue medication and adverse events. An exacerbation was defined as a worsening of asthma symptoms requiring treatment with systemic corticosteroids and increased doses of rescue medication, hospitalization or an emergency room visit, or an unscheduled physician visit. Short term trials (<52 weeks) were excluded.

### Data extraction

This systematic review was undertaken according to PRISMA guidelines^67^. Titles and abstracts were independently reviewed by two reviewers (T.L. and C.C.) to determine their potential relevance. Data from all studies included in this analysis were obtained during the end of the extension phases of the trial. Any disagreements were resolved by consensus with a third reviewer when necessary. In the case of unpublished reports or multiple publications, data from the most recent version were extracted. After obtaining full-text, the authors independently assessed all studies for inclusion based on the predefined criteria. If studies had partly overlapping subjects, the study with the larger sample size was selected. The quality of each trial was evaluated using the Cochrane five risk of bias domains tool.

### Data analysis

RR and 95% CIs were used to analyze the efficacy and safety of omalizumab as an add-on therapy for persistent uncontrolled allergic asthma. Heterogeneity assumptions were assessed using the I^2^ statistic. I^2^ values of 25%, 50% and 75% represented low, moderate and high heterogeneity, respectively^68^. When heterogeneity was noted, subgroup analyses were performed to seek out the source of the heterogeneity. Studies of poorer methodological quality, such as unblinded or open-label trials, may have exhibited exaggerated treatment effects. Excluding them may have resulted in increased internal validity but may also have reduced the external validity of the analysis^64^,^65^,^66^. To reconcile these issues, separate subgroup and sensitivity analyses were conducted, whereby the meta-analysis was reanalyzed, including risks of bias (low vs high), ages of patients (children vs adults and adolescents), asthma severity (severe vs moderate to severe) and intervention (omalizumab/ICS vs omalizumab/ICS + LABA), using a Mantel-Haenszel fixed-effects model and excluding unblinded or open label studies. Publication bias was determined using the funnel plot and assessed by Egger's test^69^. All analyses were performed with Review Manager (Version 5.0.1, The Cochrane Collaboration) and Stata (Version 10.0, Stata Corporation, USA). A p-value of <0.05 was considered to be statistically significant.

## Author Contributions

W.-T.L., B.-S.W. and W.-Z.X. have contributed to the design of the study, analysis and interpretation of data and drafting a part of manuscript. C.Z., Y.H. and C.C. searched the related papers and extracted data. M.-S.Y. and W.L. carried out statistical analysis and revised manuscript. Z.C. and Y.Z. prepared all figures. B.W. and H.-H.S. designed this study and revised manuscript.

## Supplementary Material

Supplementary Information Supplementary information

## Figures and Tables

**Table 1:** Characteristic of randomized controlled trials included

Source	Study design	Female/Patients (No.)	Age (y)‡	IgE (IU/ml)‡	Severity/FEV_1_ (%pred)‡	Study duration (weeks)	Exacerbation definition
**Finn 2003**^44^	**DB**				**S**	**52**	
Omalizumab		164/268	39.3	172.5	68.2		A worsening of asthma symptoms and was severe enough to require treatment with oral or intravenous corticosteroids or a doubling of the subject's baseline inhaled BDP dose.
Control		146/257	39.0	186.3	67.7		
**Lanier 2003**^45^	**DB**				**S**	**52**	
Omalizumab		150/245	68.8	173.4	68.8		Worsening of asthma requiring treatment with oral or intravenous corticosteroids or doubling of the patient's most recent BDP maintenance dose.
Control		119/215	68.2	186.2	68.2		
**Niven 2008**^46^	**OL**				**S**	**52**	
Omalizumab		86/115	38.7	NA	65.6		Asthma worsening requiring treatment with systemic corticosteroids and the ADRIs, unscheduled physician visit, or hospitalization/emergency room visit.
Control		34/49	39.3	NA	64.1		
**Buhl 2002**^47^	**DB**				**M-S**	**52**	
Omalizumab		124/254	41	220.2	70.0		Worsening of asthma requiring treatment with oral or parenteral corticosteroids or doubling of the patient's most recent BDP maintenance dose.
Control		120/299	40	204.1	70.4		
**Lanier 2009**^48^	**DB**				**M-S**	**52**	
Omalizumab		134/421	8.7	476.0	86.0		Worsening of asthma symptoms requiring doubling of baseline ICS dose and/or treatment with rescue systemic corticosteroids for 3 days.
Control		69/207	8.4	456.9	87.2		
**Busse 2011**^49^	**DB**				**M-S**	**60**	
Omalizumab		86/208	10.9	NA	92.9		A need for systemic glucocorticoids, hospitalization, or both, in accordance with a recent report by the American Thoracic Society/European Respiratory Society§.
Control		91/211	10.8	NA	92.2		

^‡^The data are shown as mean.

FEV_1_, forced expiratory volume in one second; DB, Double-blind; OL, Open-label; M, moderate; S, severe; BDP, beclomethasone dipropionate; ADRIs, annual rate of asthma deteriorationrelated incidents; NA, not available.

^§^Reddel HK, *et al*. An official American Thoracic Society/European Respiratory Society statement: asthma control and exacerbations: standardizing endpoints for clinical asthma trials and clinical practice. *Am J Respir Crit Care Med* **180**, 59–99 (2009).

**Table 2:** Risk of bias of the included studies

Study	Sequence generation	Allocation concealment	Data collection blinded	Complete outcome data	Selective outcome reporting
Finn et al^44^	No	Yes	Yes	Yes	Yes
Lanier et al^45^	No	Yes	Yes	Yes	Yes
Niven et al^46^	Yes	No	Yes	Yes	Yes
Buhl et al^47^	No	Yes	Yes	Yes	Yes
Lanier et al^48^	Yes	Yes	Yes	Yes	Yes
Busse et al^49^	No	Yes	Yes	Yes	Yes

**Table 3:** Results of subgroup and sensitivity analyses from a meta-analyses of randomized controlled trials

Trials	Asthma exacerbation^44^,^45^,^46^,^47^,^48^,^49^	Withdrew ICS completely^45^,^47^	Change in GFTE score^44^,^48^,^49^	AQLQ ≥ 1.5^44^,^46^	Adverse events^45^,^47^,^48^,^49^
	←RR (95%CI), P value→				
**All trials**^44^,^45^,^46^,^47^,^48^,^49^	0.63 [0.55, 0.71] <0.0001	1.86 [1.51, 2.29] <0.0001	1.54 [1.38, 1.72] <0.00001	2.08 [1.03, 4.20] = 0.04	0.97 [0.93, 1.01] = 0.11
**Subgroup analyses**					
**Risk of bias**					
Low^46^,^48^	0.57 [0.43, 0.74] <0.0001	–	1.42 [1.24, 1.62] <0.00001	3.23 [1.58, 6.59] = 0.001	0.96 [0.92, 1.01] = 0.12
High^44^,^45^,^47^,^49^	0.64 [0.55, 0.75] <0.00001	1.86 [1.51, 2.29] <0.0001	1.65 [1.45, 1.87] <0.00001	1.57 [1.23, 2.01] = 0.0003	0.96 [0.88, 1.06] = 0.44
**Age of patients**					
Adolescents and adults^44^,^45^,^46^,^47^	0.65 [0.56, 0.76] <0.00001	1.86 [1.51, 2.29] <0.0001	1.60 [1.30, 1.97] <0.00001	2.08 [1.03, 4.20] = 0.04	0.99 [0.93, 1.07] = 0.54
Children^48^,^49^	0.41 [0.29, 0.58] <0.00001	–	1.53 [1.30, 1.80] <0.00001	–	0.91 [0.75, 1.12] = 0.06
**Asthma severity**					
Moderate-sever^44^,^45^,^46^	0.68 [0.55, 0.84] = 0.0004	2.37 [1.17, 4.78] = 0.02	1.60 [1.30, 1.97] <0.00001	2.08 [1.03, 4.20] = 0.04	1.01 [0.93, 1.09] = 0.88
Severe^47^,^48^,^49^	0.58 [0.49, 0.69] <0.00001	1.82 [1.46, 2.26] <0.0001	1.53 [1.30, 1.80] <0.00001	–	0.95 [0.89, 1.02] = 0.28
**Intervention**					
Omalizumab/ICS^44^,^45^,^47^,^48^	0.63 [0.50, 0.80] = 0.0002	1.86 [1.51, 2.29] <0.0001	1.49 [1.33, 1.66] <0.00001	1.57 [1.23, 2.01] = 0.0003	0.98 [0.93, 1.02] = 0.32
Omalizumab/ICS + LABA^46^,^49^	0.59 [0.49, 0.72] <0.00001	–	1.68 [1.43, 1.97] <0.00001	3.23 [1.58, 6.59] = 0.001	0.83 [0.67, 1.04] = 0.10
**Sensitivity analyses**					
Open label^46^	0.60 [0.44, 0.81] = 0.001	–	–	3.23 [1.58, 6.59] = 0.001	–
Double-blinded^44^,^45^,^47^,^48^,^49^	0.63 [0.54, 0.73] <0.0001	1.86 [1.51, 2.29] <0.0001	1.54 [1.38, 1.72] <0.00001	1.57 [1.23, 2.01] = 0.0003	0.97 [0.93, 1.01] = 0.11
Fixed-effects model^44^,^45^,^46^,^47^,^48^,^49^	0.62 [0.55, 0.71] <0.00001	1.88 [1.52, 2.33] <0.0001	1.52 [1.37, 1.68] <0.00001	1.77 [1.40, 2.24] <0.00001	0.96 [0.91, 1.01] = 0.08

RR, relative risk; CI, confidence interval; ICS, included inhaled corticosteroid; GETE, Global Evaluation of Treatment Effectiveness; AQLQ, Asthma Quality of Life Questionnaire; LABA, long-acting beta2-agonists.

**Table 4:** Summary of cost-effectiveness trials evaluating omalizumab add-on therapy *vs* stander therapy included in the systematic review

Source	Country	Target population	Outcomes (ICER)§	Comments
**Brown 2007**^50^	Canada	Uncontrolled severe persistent asthma despite high dose ICS and LABA	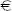 821,000/QALY (£646,783/QALY)	Omalizumab add-on therapy in patients with severe persistent asthma was cost-effective
**Campbell 2010**^51^	USA	Moderate to severe persistent asthma uncontrolled with ICS	$287,200/QALY (£176,369/QALY)	Adding omalizumab to usual care improves QALYs at an increase in direct medical costs. The value increases when omalizumab response is used to guide long-term treatment
**Dewilde 2006**^52^	Sweden	Uncontrolled severe persistent asthma despite high dose ICS and LABA	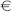 56,091/QALY (£44,188/QALY)	Omalizumab provided cost offsets, improves quality of life and may have an attractive ICER in treating the severe allergic asthma population
**Dal Negro 2011**^53^	Italy	Severe and resistant asthma despite treatment with high does ICS and LABA	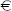 26,000/QALY (£20,482/QALY)	Omalizumab added to an optimized therapy significantly improves clinical outcomes in persistent allergic asthma. Costs also increased, but proved justified by health benefits achieved
**Nooten 2013**^54^	Netherlands	Uncontrolled allergic asthma despite treatment with high does ICS and LABA	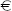 38,371/QALY (£30,228/QALY)	Non-clinical trial experience with omalizumab supported the finding of fewer exacerbations in the allergic asthma population while treated with omalizumab, and therapy was found to continue to have an attractive cost-effectiveness ratio
**Oba 2004**^55^	USA	Moderate to severe persistent asthma uncontrolled with ICS	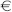 378 (£297)/0.5-point AQLQ increase	Omalizumab was clearly more expensive than other controller medications in patients with moderate allergic asthma. However, it could be cost saving if it was used in nonsmoking patients despite maximal asthma therapy
**Wu 2007**^56^	USA	Severe persistent asthma received ICS plus quick relievers (e.g. SABA)	$821,000/QALY (£504,176/QALY)	Omalizumab is not cost-effective for most patients with severe asthma. It is especially important that clinicians explore alternative medications for asthma before initiating omalizumab.

^§^Conversion to £ uses the rate of: 1 dollar = £0.6141 and 1 euro = £0.7878 (19 Sep 2014).

ICS, inhaled corticosteroid; LABA, long-acting beta2-agonists; AQLQ, Asthma Quality of Life Questionnaire; QALY, quality-adjusted life year; ICER, incremental cost-effectiveness ratio.
