# Supplementary material for: Long-term efficacy and safety of omalizumab in patients with persistent uncontrolled allergic asthma: a systematic review and meta-analysis
Source: Sci Rep. 2015 Feb 3;5:8191. doi: 10.1038/srep08191 (PMC4314644; doi:10.1038/srep08191)
Supplement: Supplementary Information [file srep08191-s1.doc]

**TITLE PAGE**

**Long-term efficacy and safety of omalizumab in patients with persistent uncontrolled allergic asthma: a systematic review and meta-analysis**

Tianwen Lai^1, 2^*, Shaobin Wang^1^*, Zhiwei Xu^1^, Chao Zhang^1^, Yun Zhao^1^, Yue Hu^1^, Chao Cao^1^, Songmin Ying^1,4^, Zhihua Chen^1^, Wen Li^1^, Bin Wu^2^ & Huahao Shen^1,3^

1. Department of Respiratory and Critical Care Medicine, Second Affiliated Hospital, Institute of Respiratory Diseases, Zhejiang University School of Medicine, Hangzhou, China.

2. Department of Respiratory and Critical Care Medicine, Affiliated Hospital, Institute of Respiratory Diseases, Guangdong Medicine College, Zhanjiang, China.

3. State Key Lab. for Respiratory Diseases, Guangzhou, China.

4. Department of Pharmacology, Zhejiang University School of Medicine, Hangzhou, China.

Correspondence and requests for materials should be addressed to Huahao Shen (huahaoshen@163.com) and Bin Wu (wubin621011@126.com).

*These authors contributed equally to this work.

| **Supplemental table 1. Characteristic of the main trials excluded** | | | | | | |
| --- | --- | --- | --- | --- | --- | --- |
| **Source**  **(treatment group)** | **Patients**  **(n)** | **Age^‡^ (y)** | **IgE*(IU/ml)** | **Asthma severity/**  **FEV_1_ (% pred)*** | **Study duration**  **(weeks)** |  |
| **Bardelas 2012**^[23]^ |  |  |  | **M-S** | **26** | |
| Omalizumab | 136 | 41.9 | 183.6 (126.1) | 74.5 (17.5) |  | |
| Control | 135 | 40.7 | 180.5 (135.3) | 76.5 (17.0) |  | |
| **Bousquet 2011**^[24]^ |  |  |  | **S** | **32** | |
| Omalizumab | 272 | 45.6 | 233.3 (153.43) | 63.0 (12.41) |  | |
| Control | 128 | 45.7 | 231.4 (149.86) | 61.1 (13.37) |  | |
| **Busse 2001**^[25]^ |  |  |  | **M-S** | **28** | |
| Omalizumab | 268 | 39.3 | 172.5 (20–860) | 68.2 (14.9) |  | |
| Control | 257 | 39.0 | 186.3 (21–702) | 67.7 (14.3) |  | |
| **Chanez 2010**^[26]^ |  |  |  | **S** | **16** | |
| Omalizumab | 20 | 45.7 | 246.5 (29.0–384.0) | 61.3 (14.83) |  |  |
| Control | 11 | 50.6 | 216.0 (31.0–602.0) | 66.6 (11.38) |  |  |
| **Hanania 2011**^[27]^ |  |  |  | **S** | **48** |  |
| Omalizumab | 427 | 43.7 | 178.7 (134.5) | 65.4 (15.2) |  |  |
| Control | 421 | 45.3 | 175.1 (133.7) | 64.4 (13.9) |  |  |
| **Holgate 2004**^[28]^ |  |  |  | **S** | **32** |  |
| Omalizumab | 126 | 41.1 | 266.8 (218.0) | 62.9 (17.5) |  |  |
| Control | 120 | 40.5 | 265.7 (190.2) | 66.0 (20.2) |  |  |
| **Hoshino 2012**^[29]^ |  |  |  | **S** | **24** |  |
| Omalizumab | 14 | 59.2 | 248.0 (170.3) | 65.3 (13.9) |  |  |
| Control | 16 | 51.2 | 282.0 (192.5) | 68.4 (12.2) |  |  |
| **Humbert 2005**^[30]^ |  |  |  | **S** | **28** |  |
| Omalizumab | 209 | 43.4 | 197.6 (145.18) | 61.0 (14.42) |  |  |
| Control | 210 | 43.3 | 189.6 (153.14) | 61.6 (13.83) |  |  |
| **Kopp 2009**^[31]^ |  |  |  | **M** | **18** |  |
| Omalizumab | 70 | 30 | 145 (20.1–641.3) | 98 (76–120) |  |  |
| Control | 70 | 33 | 133.5 (31.6–48.9) | 98 (76–120) |  |  |
| **Lemanske 2002^[32]^** |  |  |  | **M** | **28** |  |
| Omalizumab | 225 | 9.4 | 348 (20–1269) | 84 (49–129) |  |  |
| Control | 109 | 9.5 | 323 (29–1212) | 85 (43–116) |  |  |
| **Massanari 2009**^[33]^ |  |  |  | **M-S** | **28** |  |
| Omalizumab | 76 | 14.2 | 353.0 (233.44) | NA |  |  |
| Control | 70 | 14.2 | 291.6 (190.60) | NA |  |  |
| **Massanari 2010**^[34]^ |  |  |  | **M-S** | **26** |  |
| Omalizumab | 136 | 38.2 | 185.70 (138.331) | 88.1 (11.64) |  |  |
| Control | 139 | 38.2 | 167.76 (137.628) | 86.1 (11.18) |  |  |
| **Milgrom 2001**^[35]^ |  |  |  | **M-S** | **32** |  |
| Omalizumab | 225 | 9.4 | 348 (20–1269) | 84 (49–129) |  |  |
| Control | 109 | 9.5 | 323 (29–1212) | 85 (43–116) |  |  |
| **Milgrom 2011**^[36]^ |  |  |  | **M-S** | **28** |  |
| Omalizumab | 624 | 8.8 | 435.9 (323.24) | 85.2 (16.87) |  |  |
| Control | 302 | 8.6 | 414.7 (323.97) | 86.6 (17.09) |  |  |
| **Ohta 2009**^[37]^ |  |  |  | **M-S** | **16** |  |
| Omalizumab | 151 | 48.8 | 261.4 (165.31) | 74.06 (19.912) |  |  |
| Control | 164 | 49.2 | 246.7 (157.75) | 75.81 (20.888) |  |  |
| **Rubin 2012**^[38]^ |  |  |  | **S** | **20** |  |
| Omalizumab | 78 | 43.8 | 218.8 (165) | NA |  |  |
| Control | 38 | 45.2 | 249.2 (197) | NA |  |  |
| **Siergiejko 2011**^[39]^ |  |  |  | **S** | **32** |  |
| Omalizumab | 59 | 45.2 | 206.7 (140.50) | 61.3 (13.64) |  |  |
| Control | 23 | 45.6 | 274.9 (167.93) | 60.7 (11.72) |  |  |
| **Solèr 2001**^[40]^ |  |  |  | **M-S** | **28** |  |
| Omalizumab | 274 | 40.0 | 223.1 (21–785) | 69.8 (30-112) |  |  |
| Control | 272 | 39.0 | 205.6 (22–814) | 69.9 (22-109) |  |  |
| **Vignola 2004**^[41]^ |  |  |  | **M-S** | **28** |  |
| Omalizumab | 209 | 38.3 | NA | 76.9 (15.72) |  |  |
| Control | 196 | 38.5 | NA | 79.4 (17.46) |  |  |
| **Zielen 2013**^[42]^ |  |  |  | **M** | **16** |  |
| Omalizumab | 34 | 36 | 1325.38 (312.4) | 94.28 (17.1) |  |  |
| Control | 16 | 34 | 598.58 (592.9) | 96.68 (15.4) |  |  |
| ^‡^ mean baseline, *mean (SD or range).  FEV_1_, forced expiratory volume in 1second; M, moderate; S, severe; NA, not available. | | | | | | |

| **Supplemental table 2. Analysis of other outcomes (Omalizumab vs control)** | | | | | | |
| --- | --- | --- | --- | --- | --- | --- |
|  |  |  | **Effects** | | |  |
| **Outcome measure** | **Omalizumab effect** | **No.**  **(O/C)** | **O (Δ)** | **C (Δ)** | **Difference between groups: *P* value** | **Comments** |
| **Asthma symptom score** |  |  |  |  |  |  |
|  | **Niven 2008** ^[46]^ ↑↑ | 115/49 | NA | NA | *p* < 0.05 | At 1 year, asthma score was significantly improved in the omalizumab group compared with control. |
|  | **Liner 2009** ^[48]^ ↑↑ | 421/207 | NA | NA | *p* < 0.01 | Nocturnal asthma symptom score, -0.63 [0.72] vs -0.50 [0.71]. |
| **Pulmonary function** |  |  |  |  |  |  |
| **Lanier 2003** ^[45]^ |  | 245/215 |  |  |  |  |
|  | FEV_1_, L ↔ |  | NA | NA | *P* = 0.16 | The corresponding between-group differences in FEV_1_ at weeks 52 were 52 ml. |
| **Niven 2008** ^[46]^ | FEV_1_, L ↑↑ | 115/49 | Δ = + 0.19 | NA | *P* < 0.05 | Throughout the 1-year treatment period representing a between-group difference of 320 ml. |
| **Buhl 2002** ^[47]^ |  |  |  |  |  |  |
|  | FEV_1_, %predicted ↔ | 254/299 | NA | NA | NA | No statistically significant differences in FEV_1_ were seen between the treatment groups at any time point during the extension. |
| **Busse 2011** ^[49]^ |  |  |  |  |  |  |
|  | FEV_1_, %predicted ↔ | 208/211 | NA | NA | *p* = 0.30 | No statistically significant differences in FEV_1_ were seen between two groups during follow-up. |
| ↑↑ Omalizumab better than (with statistical significance); ↑ Omalizumab better than control (without statistical significance); ↔ Omalizumab comparable with control. O, Omalizumab; C, Control; Δ, The mean change from baseline; NA, not available; FEV_1_, forced expiratory volume in 1 second. | | | | | | |
